# Supplementary material for: COZOID: contact zone identifier for visual analysis of protein-protein interactions
Source: BMC Bioinformatics. 2018 Apr 6;19:125. doi: 10.1186/s12859-018-2113-6 (PMC5889581; doi:10.1186/s12859-018-2113-6)
Supplement: Supplementary file 2 — Software build. Executable binary file of the software tool. (ZIP 118,784 kb) [file 12859_2018_2113_MOESM2_ESM.zip › cozoid/java/docs/beansbinding-1.2.1-doc.zip!/org/jdesktop/el/impl/lang/ELArithmetic.BigDecimalDelegate.html]

ELArithmetic.BigDecimalDelegate (Beans Binding)


---


|  |  |  |
| --- | --- | --- |
| **Overview** \| **Package** \| **Class** \| **Use** \| **Tree** \| **Deprecated** \| **Index** \| **Help** | |  |
| **PREV CLASS**   **NEXT CLASS** | **FRAMES**    **NO FRAMES**     **All Classes** |  |
| SUMMARY: NESTED \| FIELD \| CONSTR \| METHOD | DETAIL: FIELD \| CONSTR \| METHOD |  |


---


## org.jdesktop.el.impl.lang Class ELArithmetic.BigDecimalDelegate

```
java.lang.Object
  org.jdesktop.el.impl.lang.ELArithmetic
      org.jdesktop.el.impl.lang.ELArithmetic.BigDecimalDelegate
```

**Enclosing class:**: ELArithmetic

---

``` public static final class ELArithmetic.BigDecimalDelegate extends ELArithmetic ```

---

**Nested Class Summary**

**Nested classes/interfaces inherited from class org.jdesktop.el.impl.lang.ELArithmetic**

`ELArithmetic.BigDecimalDelegate, ELArithmetic.BigIntegerDelegate, ELArithmetic.DoubleDelegate, ELArithmetic.LongDelegate`


**Field Summary**

**Fields inherited from class org.jdesktop.el.impl.lang.ELArithmetic**

`BIGDECIMAL, BIGINTEGER, DOUBLE, LONG`


**Constructor Summary**

`ELArithmetic.BigDecimalDelegate()`


| **Method Summary** | |
| --- | --- |
| `protected  java.lang.Number` | `add(java.lang.Number num0, java.lang.Number num1)` |
| `protected  java.lang.Number` | `coerce(java.lang.Number num)` |
| `protected  java.lang.Number` | `coerce(java.lang.String str)` |
| `protected  java.lang.Number` | `divide(java.lang.Number num0, java.lang.Number num1)` |
| `boolean` | `matches(java.lang.Object obj0, java.lang.Object obj1)` |
| `protected  java.lang.Number` | `mod(java.lang.Number num0, java.lang.Number num1)` |
| `protected  java.lang.Number` | `multiply(java.lang.Number num0, java.lang.Number num1)` |
| `protected  java.lang.Number` | `subtract(java.lang.Number num0, java.lang.Number num1)` |

**Methods inherited from class org.jdesktop.el.impl.lang.ELArithmetic**

`add, coerce, divide, isNumber, isNumberType, mod, multiply, subtract`

**Methods inherited from class java.lang.Object**

`clone, equals, finalize, getClass, hashCode, notify, notifyAll, toString, wait, wait, wait`

**Constructor Detail**

### ELArithmetic.BigDecimalDelegate

```
public ELArithmetic.BigDecimalDelegate()
```


**Method Detail**

### add

```
protected java.lang.Number add(java.lang.Number num0,
                               java.lang.Number num1)
```

:   **Specified by:**: `add` in class `ELArithmetic`

---


### coerce

```
protected java.lang.Number coerce(java.lang.Number num)
```

:   **Specified by:**: `coerce` in class `ELArithmetic`

---


### coerce

```
protected java.lang.Number coerce(java.lang.String str)
```

:   **Specified by:**: `coerce` in class `ELArithmetic`

---


### divide

```
protected java.lang.Number divide(java.lang.Number num0,
                                  java.lang.Number num1)
```

:   **Specified by:**: `divide` in class `ELArithmetic`

---


### subtract

```
protected java.lang.Number subtract(java.lang.Number num0,
                                    java.lang.Number num1)
```

:   **Specified by:**: `subtract` in class `ELArithmetic`

---


### mod

```
protected java.lang.Number mod(java.lang.Number num0,
                               java.lang.Number num1)
```

:   **Specified by:**: `mod` in class `ELArithmetic`

---


### multiply

```
protected java.lang.Number multiply(java.lang.Number num0,
                                    java.lang.Number num1)
```

:   **Specified by:**: `multiply` in class `ELArithmetic`

---


### matches

```
public boolean matches(java.lang.Object obj0,
                       java.lang.Object obj1)
```

:   **Specified by:**: `matches` in class `ELArithmetic`


---


|  |  |  |
| --- | --- | --- |
| **Overview** \| **Package** \| **Class** \| **Use** \| **Tree** \| **Deprecated** \| **Index** \| **Help** | |  |
| **PREV CLASS**   **NEXT CLASS** | **FRAMES**    **NO FRAMES**     **All Classes** |  |
| SUMMARY: NESTED \| FIELD \| CONSTR \| METHOD | DETAIL: FIELD \| CONSTR \| METHOD |  |


---
